# Impact of daily octenidine skin washing versus nonwashing on antiseptic tolerance of coagulase-negative staphylococci in two neonatal intensive care units with different skin cleansing practices

**DOI:** 10.1016/j.infpip.2024.100344

**Published:** 2024-02-03

**Authors:** Heather Felgate, Charlotte Quinn, Ben Richardson, Carol Hudson, Dheeraj Sethi, Sam Oddie, Paul Clarke, Mark A. Webber

**Affiliations:** a Quadram Institute Bioscience (QIB), Norwich Research Park, Norwich, UK; b Norwich Medical School, University of East Anglia (UEA), Norwich, UK; c Neonatal Unit, Bradford Royal Infirmary, Bradford, UK; d Neonatal Unit, Norfolk and Norwich University Hospitals NHS Foundation Trust, Norwich, UK

**Keywords:** Late onset infection, Antimicrobial resistance, Disinfection, Bathing, Antiseptics

## Abstract

**Background:**

There is wide variation in practices regarding routine bathing/washing of babies in neonatal intensive care units (NICUs). Evidence is lacking as to the benefit of routine antiseptic washes for reducing infection. We aimed to compare the antiseptic tolerance of Coagulase Negative Staphylococci (CoNS) within two UK NICUs with very different approaches to skin washing.

**Methods:**

We compared antiseptic susceptibility of CoNS isolated from skin swabs of neonates admitted to the Norfolk and Norwich University Hospital (NNUH) NICU in December 2017–March 2018 with those isolated in the Bradford Royal Infirmary (BRI) NICU in January–March 2020. The NNUH does not practise routine whole-body washing whereas BRI practises daily whole-body washing from post-menstrual age 27 weeks using Octenisan wash lotion (0.3% octenidine; 1 minute contact time before washing off with sterile water). A total of 78 CoNS isolates from BRI and 863 from the NNUH were tested for susceptibility against the antiseptics octenidine (OCT) and chlorhexidine (CHX).

**Results:**

Isolates from the BRI with practice of routine washing did not show increased antiseptic tolerance to OCT or CHX. Isolates from the NNUH which does not practise routine whole-body washing and rarely uses octenidine, were comparatively less susceptible to both CHX and OCT antiseptics.

**Conclusions:**

Daily whole-body skin washing with OCT does not appear to select for CoNS isolates that are antiseptic tolerant towards OCT and CHX. There remains considerable uncertainty about the impact of different antiseptic regimes on neonatal skin microbiota, the benefit of routine washing, and the development of antiseptic tolerance in the NICU.

## List of abbreviations

BRI: Bradford Royal Infirmary
CoNS: Coagulase Negative Staphylococci
CHX: Chlorhexidine gluconate
CVC: Central Venous Catheter
MIC: Minimum Inhibitory Concentration
NICU: Neonatal Intensive Care Unit
NNUH: Norfolk and Norwich University Hospital
OCT: Octenidine

## Introduction

Infection is common among premature and very low birth weight (<1500 g) infants, due to the immaturity of immune system, skin and mucosal barriers [1,2]. Late-onset infection, occurring after the first 72 hours from birth, is usually nosocomial and caused by organisms from the skin microbiota or hospital environment [3]. Within neonatal intensive care units (NICUs), invasive procedures are essential for management but indwelling catheters are a major source of infection [4]. Coagulase-negative staphylococci (CoNS) are common skin commensals, which cause up to 80%–90% of late-onset sepsis in NICUs [5]. Catheter-related sepsis can be life-threatening and cause permanent lifelong injury and disability in survivors, including cerebral palsy and other adverse neurodevelopmental problems [3,[6], [7], [8], [9]].

CoNS rapidly colonise the skin of infants after birth, with the most prevalent species being *S. epidermidis, S. haemolyticus*, *S. warneri,* and S. *capitis* [10,11]. Disruption of the skin barrier by insertion of intravascular devices can lead to colonisation of the outside of these devices (such as central venous catheters; CVCs). This can then lead to bloodstream and catheter-related infections, which can in turn lead to systemic infection and neonatal sepsis [4,5,12,13].

Antiseptics are used before procedures to minimise the risk of infection at the site of the anticipated skin breach [14]. In addition, within both adult and paediatric populations, there is evidence that regular bathing using antiseptics including chlorhexidine gluconate (CHX) can reduce the number of hospital-acquired infections within intensive care [[15], [16], [17]]. However this has not been observed for CHX-based body washing in neonates [18]. Whilst there are national evidence-based guidelines for antiseptic use in children, there is no UK standardised guidance in place for preferred antiseptics infants who are less than 2 months old, [19] nor regarding routine washing or bathing practices. A national UK survey of tertiary-level NICUs done in 2019-20 found that 3/57 (5%) units virtually never bathe their NICU babies, while 44/57 (77%) bathe regularly once the baby is out of NICU/high-dependency; of those practising bathing, 67% used tap water and 33% used sterile water; 14% were using adjunct antiseptic agents or cleansing products [20]. There is presently therefore a wide range of practices in operation regarding antiseptic choices for pre-procedural local skin disinfection prior to catheterisation [21,22], and also in respect of body washing and bathing practices [20]. The most common antiseptics used in Europe are alcohol-and aqueous-based CHX, octenidine (OCT), and povidone iodine, but there are very wide variations in CHX concentrations being used in the NICU (0.015–2%) [[21], [22], [23]].

CHX is a cationic bisguanide with a broad spectrum of antimicrobial activity [24]. It has been shown that regular bathing with CHX significantly reduces the bacterial skin burden in neonates [25], however, the duration of this reduction and subsequent impact on reducing neonatal bloodstream infections and sepsis, is much less clearcut, varying form 36–65 % reduction in bloodstream infections [8,26,27]. OCT is a bis-pyridine compound which also has a broad spectrum of antimicrobial activity. Very few studies have examined the use of OCT within a neonatal population, however there is evidence that it is effective at reducing hospital acquired infection amongst adults and older children [[28], [29], [30]]. In some centres OCT has been introduced as a whole-body wash [20], as it is reportedly mild and suitable for patients with vulnerable skin.

In this study, we aimed to compare antiseptic susceptibility amongst two panels of isolates of CoNS from UK NICUs with very disparate bathing policies: Bradford Royal Infirmary (BRI) carries out daily whole-body washes for infants during their admission using an OCT-based antiseptic, while the Norfolk and Norwich University Hospital (NNUH) does not routinely whole-body wash or bathe infants at all, either with antiseptic or with water, between admission and discharge. Both NICUs use CHX for pre-procedural topical antisepsis. Previous work has shown that use of CHX alone can increase biocide resistance [31], however the effect of using both OCT and CHX has yet to be investigated.

Our hypothesis was that CoNS isolates from the skin in BRI infants who undergo daily whole-body skin washing with OCT would show higher tolerance to OCT compared with infants from NNUH which did not practise routine daily washing and which rarely used OCT. Thus the primary outcome was to determine whether routine washing of babies with OCT impacts the tolerance to OCT and abundance of CoNS isolated from skin. Both OCT and CHX are structurally similar (biguanide compounds) antiseptics that are commonly used for cleansing in hospitals. A secondary aim was to determine whether exposure to OCT or CHX increases tolerance to either antiseptic, by comparing isolates obtained from the BRI unit where regular washing of babies with OCT may impact CoNS tolerance to CHX, compared with those isolates from the NNUH where infants who were not routinely whole-body washed but are frequently exposed to CHX.

## Methods

### Study sites and routine cleansing practices

This was a two-site observational study which involved two similar sized tertiary-level UK hospital NICUs which each cater for just under 6000 deliveries per year. Both provide intensive care to neonates of all gestational ages from 22 weeks to term. NNUH does not practise whole-body skin cleansing routinely on any infant at any time during their whole NICU admission.

The NICU at BRI practises daily whole-body wash downs of all infants ≥27 weeks' gestation using Octenisan® wash lotion (Schülke & Meyr), containing 0.3% octenidine dihydrochloride, applied to the skin using cotton wool, then washed off after ∼1 minute using sterile water (a local protocol in use since 2007). All infants born ≥27 weeks' gestational age were included in the daily skin disinfection regime. Per hospital policy, infants with broken/immature skin were excluded from the washing regime (therefore OCT-naïve), as were those born at <27 weeks' gestation until they had reached 27 weeks' post-menstrual age.

### Routine NICU practices in place at the time of CoNS isolation

In addition to neonatal washing with OCT or localised sterilising procedures, in both centres, tap water used by parents and carers for routine hand washing was filtered to 0.2 μm, and sterile water was used for any direct skin cleansing. Both centres routinely used CHX-based antiseptics (0.015%–2% CHX in 70% isopropanolol) for pre-procedural skin disinfection before the insertion of indwelling catheters, including for peripheral and central venous catheters, and peripheral and umbilical arterial catheters. OCT was only used exceptionally in NNUH centre, in the form of Octenisan antiseptic wash (Schülke & Mayr, GmbH), for very occasional decolonisation of individual methicillin-resistant Staphylococcus aureus-positive infants. Both the NNUH and the BRI used CHX-based wipes (0.1–2% CHX) for surface and environmental cleaning, such as incubators and equipment.

### Isolate collection

As part of a previous prospective surveillance study involving the Quadram Institute and NNUH, done in December 2017 to March 2018 [31], a panel of 863 CoNS were isolated from skin swabs taken at the NNUH NICU; in total 123 babies were swabbed. Swabs were taken on admission and once weekly from each baby throughout their NICU stay from the ear, axilla, groin and rectum [31]. All infants currently or newly admitted had been eligible for swab collection study during the study period, regardless of birth gestational age or expected duration of stay.

For the present study, infants admitted to the BRI NICU prospectively had skin swabs taken on admission and then once weekly for their duration of stay, over a period of 8 weeks (between January and March 2020). A single charcoal swab (Amies Charcoal Transport Swab) was used to take a body sweep, incorporating the ear, neck, an axilla, umbilical area, and groin. The swabbing was typically carried out 12–16 hours after the daily washing had occurred. Swabs were stored locally at 4 ^o^C. Batches were securely packaged and posted to the Quadram Institute Bioscience (QIB), Norwich, every 3 weeks, where they were stored at 4 ^o^C upon arrival.

A unique study ID was allotted to each infant enrolled using their anonymised code generated by the BadgerNet neonatal platform (CleverMed, UK). Birth weights, dates of admission, swab number, birth gestational age, gender of infant, birthing method, location of birth and corrected gestational age at enrolment were collected. No identifying data were transmitted out of the participating sites. Completed anonymised data were collated at QIB into a master database.

### Isolation of CoNS

Charcoal swabs from both sites were streaked on Columbia Blood Agar (CBA; Oxoid Thermo Fisher Scientific, USA), candidate CoNS were then sub-cultured on Mannitol-Salt Agar (MSA; Oxoid Thermo Fisher Scientific, USA). Isolates were tested for coagulase (Coagulase Test Slides, Millipore, Sigma), and any isolates suspected to be *Enterococci* were grown on Bile Aesculin Agar (Oxoid Thermo Fisher Scientific, USA). Finally, catalase tests were used with 20% hydrogen peroxide. Isolates considered to be CoNS based on the phenotyping above were saved and given a unique study number.

### Antimicrobial susceptibility testing

The minimum inhibitory concentrations (MICs) of OCT and CHX were determined for all isolates using the European Committee on Antimicrobial Susceptibility Testing (EUCAST) as a guideline [32]. Mueller Hinton (MH) Agar (Oxoid) was prepared with concentrations of antiseptics ranging from 0.25 μg/mL to 64 μg/mL. Overnight cultures grown in MH broth were diluted 1/10,000 and 1 μL drops were plated on to the antiseptic-containing MH Agar and incubated at 37 ^o^C for 24 hrs. Two control strains, TW20 and F77, were used throughout [31]. An MIC breakpoint of 4 μg/mL has been suggested to determine CHX resistance; no breakpoints have been proposed for OCT to date although 2 μg/mL has been used previously as an epidemiological cut off [31,33]. As the clinical breakpoints for these antiseptics are unknown, we used these cut offs as guidelines for threshold reduced antiseptic susceptibility, rather than claiming these strains as resistant.

### Statistics

Data were analysed using GraphPad (PRISM 5). Correlation analysis used nonparametric Spearman tests, one-tailed with confidence levels of 95%. The nonparametric one-tailed T-Test and the Mann-Whitney test were used to identify significant differences between MIC data with a 95% confidence level.

### Ethics and consent

Informed consent was not required from parents/guardians of infants involved in this study. All patients were treated in line with routine local infection control and surveillance practice guidelines and the study was classed, before commencement, as a surveillance study after protocol review by the NNUH research services manager. The study therefore did not require formal UK Research Ethics committee review or Health Research Authority approval because it did not meet contemporaneous criteria for NHS Research requiring such prior approvals. Patients did not undergo randomisation or any intervention beyond routine care and routine surveillance swabbing. Data were analysed and are presented anonymously.

## Results

### Isolation of CoNS from Bradford neonates

A total of 55 infants from BRI were enrolled in the study. Median birth weight was 1320 g (range: 460 g–4320 g) and birth gestational age was 30.7 weeks (range: 23.0–42.0 weeks). 30/55 (55%) were male. Delivery mode was vaginal for 35 babies, by Caesarean section for 20 babies. 45/55 (82%) were inborn, and 10 (18%) were transferred in postnatally. Post-menstrual age at enrolment was median 33.3 weeks (interquartile range: 30.0–39.0 weeks). From these 55 infants, 200 skin swabs were taken, 31 upon admission to NICU, (24 had no admission swab at point of study entry) and a further 169 taken weekly during the NICU stay. Median number of swabs taken per baby was 3 (range: 1–6; interquartile range 2–5). As expected, (due to longer NICU stay) there were strong inverse correlations with both birthweight and gestational age for number of swabs taken per baby (Kendall's rank correlation 2-sided *P*<0.0001 for both), with lightest and most premature babies having disproportionately more swabs taken in total.

After swabs were incubated, plates typically demonstrated heavy growth of both Gram-positive and Gram-negative isolates demonstrating various colony morphologies. From 33 infants a total of 180 Gram-positive isolates were identified and a total of 78 were confirmed as CoNS and retained for phenotypic testing.

### Susceptibility of Bradford CoNS isolates to OCT and CHX antiseptics

Isolates generally showed increased susceptibility to OCT and the MICs ranged between ≤0.125–1 μg/mL with the majority (48.7%) of the isolates (*N*=38) being inhibited by ≤0.125 μg/mL. A total of 34 isolates (43.4%) had a MIC of 0.25–0.5 μg/mL and the remaining 6 (7.7%) had a MIC of OCT of 1 μg/mL.

For 35 of the isolates (44.9%), the MIC of CHX was ≤0.125 μg/mL; for 29 (37.2%) it was 0.25 μg/mL; 1 isolate had a CHX MIC of 0.5 μg/mL and the remaining 12 (15.4 %) had a CHX MIC of 1 μg/mL. No isolate from BRI was above the proposed breakpoints for either antiseptic.

**Figure 1:**
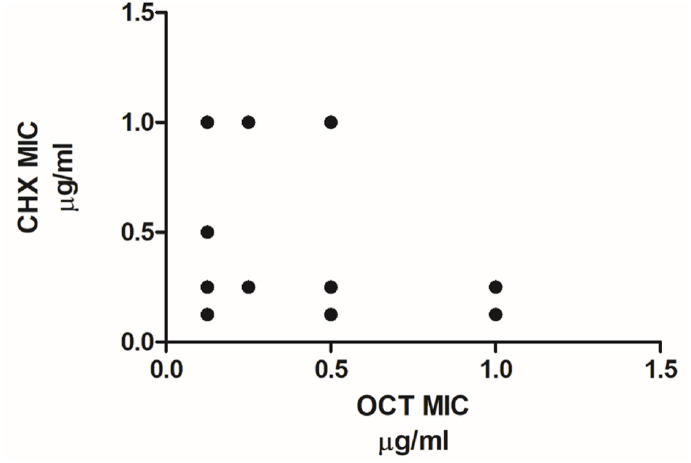
Susceptibility of isolates from BRI to OCT and CHX showed no correlation (*P*=0.4, according to Spearman test).

The CHX and OCT MIC data for each of the BRI isolates were compared against each other to determine whether there was any correlation between their susceptibility to the two agents. This analysis (Figure 1) showed no direct correlation between susceptibility to the two antiseptics (*P* = 0.4), which is similar to our previous findings for the NNUH cohort [31].

### Comparative antiseptic susceptibility of isolates from Bradford and Norwich

**Figure 2:**
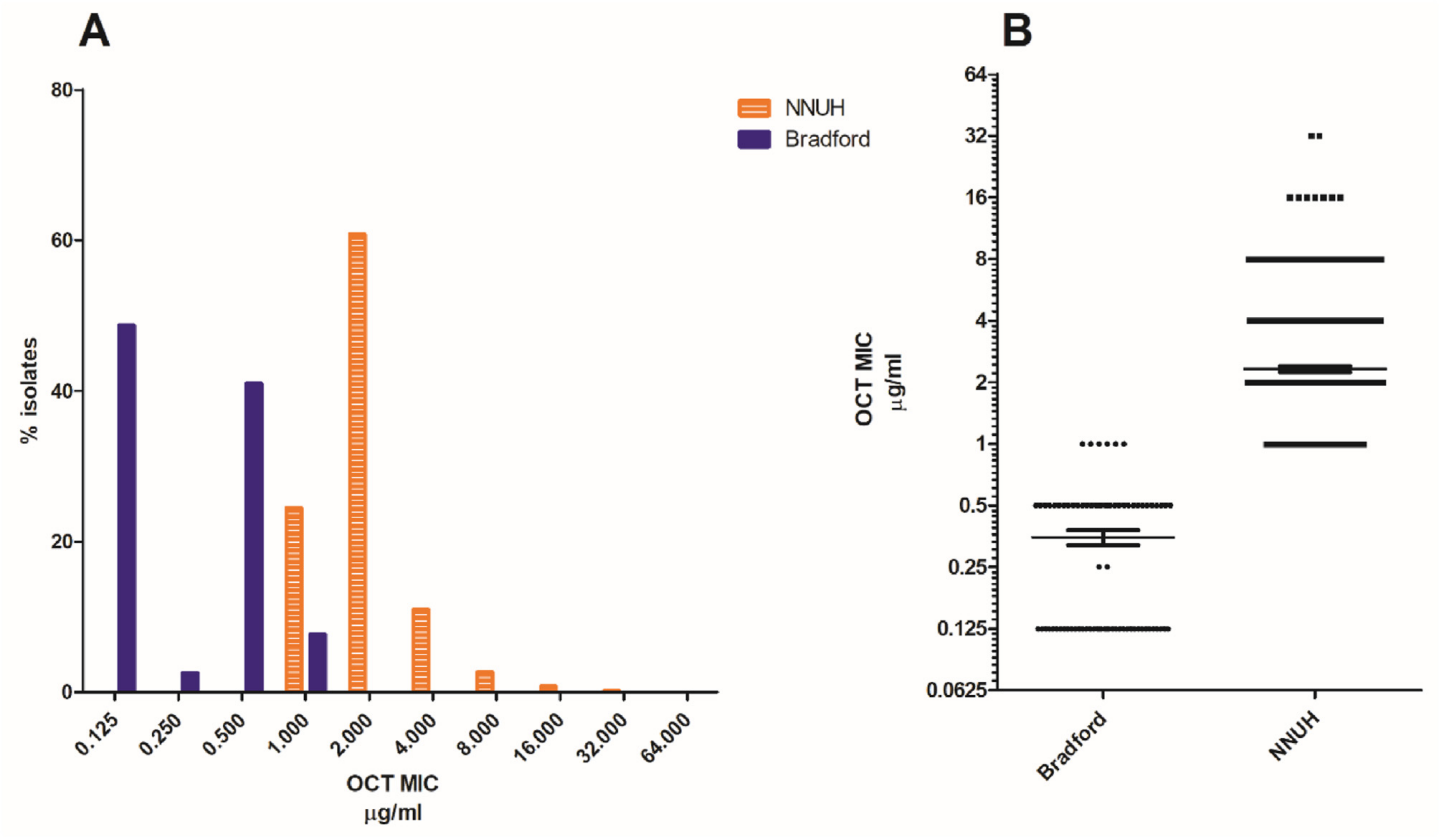
Comparison of MICs of OCT against isolates from BRI where regular daily whole-body OCT washing was in place (*N*=78) and isolates from NNUH where there was no regular washing of neonates while in NICU (*N*=863) [A]. Boxplot showing numbers of isolates with different OCT MICs from each site (∗∗∗∗ *P*= <0.0001). Thin horizontal line indicates the mean and whiskers standard error [B].

**Figure 3:**
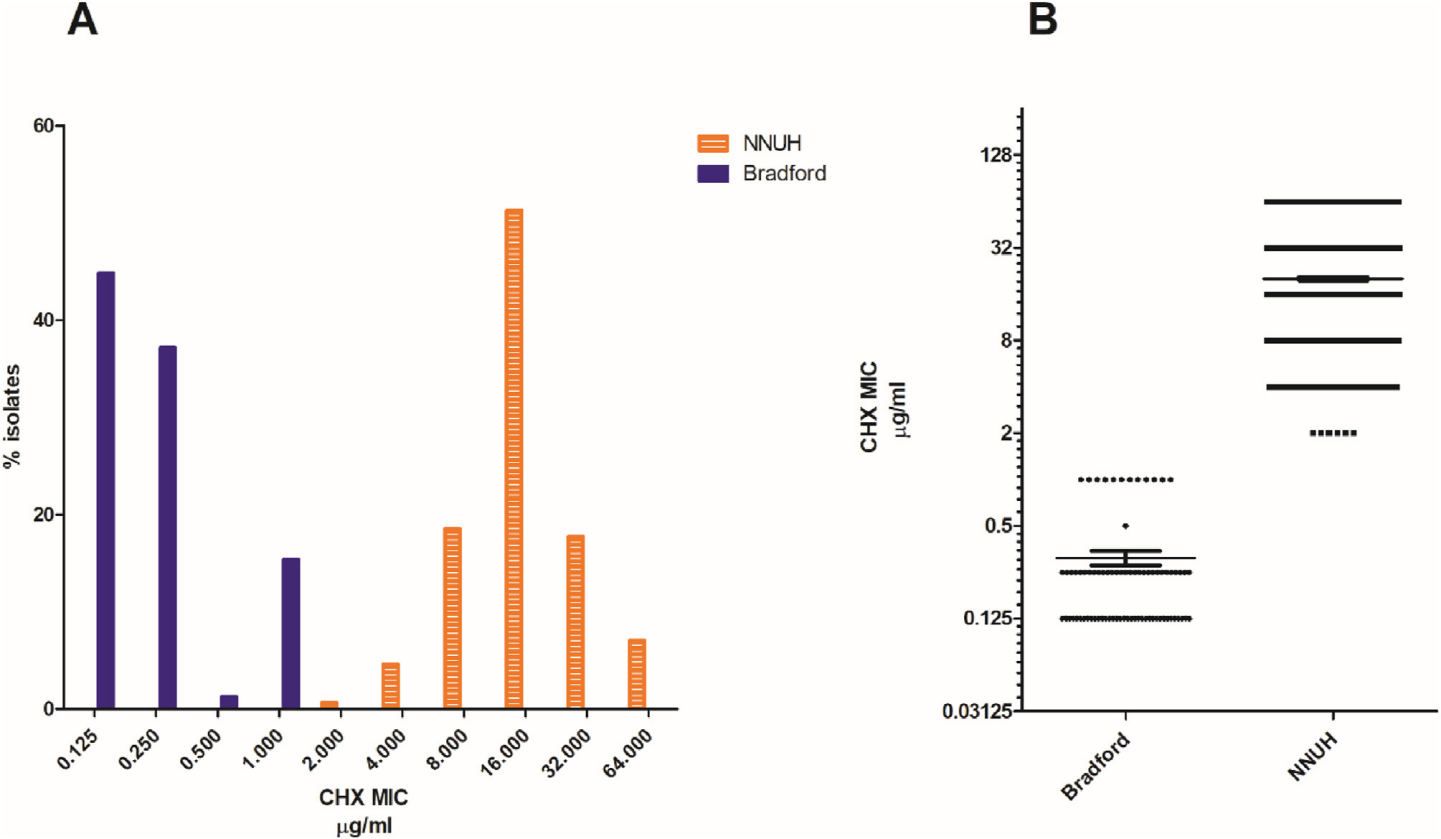
Comparison of MICs of CHX against isolates BRI where regular daily whole-body OCT washing was in place (*N*=78) and isolates from NNUH where there was no regular washing of neonates while in NICU (*N*=863) [A]. Boxplot showing numbers of isolates with different CHX MICs from each site (∗∗∗∗ *P*= <0.0001). Thin horizontal line indicates the mean and whiskers standard error [B].

Antiseptic susceptibility of the BRI isolates from the daily OCT-washed babies was compared with the panel of 863 CoNS isolated from babies in the NNUH NICU where infants were not routinely bathed. A comparison in the susceptibility profiles of the population of CoNS from BRI and NNUH showed significantly decreased susceptibility in the NNUH population to both antiseptics (Figure 2, Figure 3).

The MICs of OCT for infants from NNUH ranged between 1 and 16 μg/mL (mean of 2.319 SEM ±0.078 μg/mL), compared with a narrower range of ≤0.125 and 1 μg/mL (mean of 0.394 SEM ±0.029 μg/mL) for BRI isolates (Figure 2A). There was a significant difference in the mean MIC for OCT between Bradford and Norwich NICUs, (*P*<0.0001, Figure 2B). The MICs of CHX for NNUH isolates ranged between 2 to 64 μg/mL (mean of 20.1 SEM ±0.5 μg/mL), compared with a range of ≤0.125–1.0 μg/mL for isolates from babies at BRI (mean of 0.31 SEM ±0.04 μg/mL) (Figure 3). These data concord with previous MIC data from Sethi et al., 2021 [31]. A clear difference in the distribution of CHX susceptibility of the isolates from the two sites can be observed. There was a significant difference between the mean MIC for CHX between the BRI and NNUH isolates (*P*<0.0001, Figure 3). In total 817 (94.7 %) isolates from infants at the NNUH had a MIC for CHX greater than 4 μg/mL whereas no isolates from BRI had a MIC of CHX >1 μg/mL.

## Discussion

In this study we sought to examine antiseptic tolerance of CoNS towards CHX and OCT from two different NICUs in the UK that had very disparate practices regarding whole-body antiseptic washing of neonates. One of the main findings when looking at the antiseptic tolerance collectively across the NICUs was that there was no correlation between the MIC in CHX and OCT (Figure 1). These data suggest that use of either antiseptic should not necessarily lead to an increase in tolerance to the other.

Reassuringly, our susceptibility data do not suggest that repeated/frequent exposure to OCT selects for antiseptic tolerance on skin isolates (Figure 2, Figure 3A). Paradoxically, in fact, isolates from BRI were significantly more susceptible to both antiseptics than those from NNUH and all isolates with highest MICs were from NNUH. This is similar to our recent comparison of the NNUH NICU's panel with a panel from a NICU in Lübeck, Germany (where OCT-based rather than CHX-based antiseptics were regularly used for skin disinfection prior to catheter insertion) and again suggests CHX exposure over decades appears more likely to select for antiseptic tolerance than OCT [31]. The substantive effect of CHX may result in long lasting low residual concentrations of CHX remaining on the skin which might provide an environment for selection of tolerant mutants. It is also possible that the historic prior use in NNUH of very low CHX concentration products (0.015% CHX) for routine pre-procedural disinfection [6], has facilitated the development of CHX tolerance over a long period of time. Alternatively, CHX is more commonly incorporated in environmental cleaning wipes and products than OCT which may also reflect a greater selective pressure for isolates with decreased tolerance.

Of interest, there appeared to be more variability in bacterial diversity observed in swabs collected at BRI compared with those from NNUH. Although this was based on observational evidence, it suggests that the daily OCT washing regime did not sterilise neonatal skin beyond the short term, with Gram Positive and Negative bacteria being quickly reinstated soon after washing (Supplementary Table 1). This observation is in line with a previous study on CHX bathing in the NICU where an initial decrease in the bacterial skin burden after application was observed, with the baseline levels of bacterial numbers being returned to after approximately 72 hours [25]. It is also conceivable that BRI's local practice of washing off the OCT 1 minute after application has limited the apparent antiseptic efficacy of OCT, contributing to the rapid restoration of skin bacteria.

## Strengths and limitations

This is the first study to assess microbiological impacts from practising routine daily washing of babies with an antiseptic within the NICU environment compared to not routinely washing. This study also suggests daily OCT washing does not select for decreased antiseptic susceptibility in CoNS in the NICU, and assessment of the bacterial burden of plates shows daily OCT washing appears to have a limited temporal impact on reduction in skin microbiota. Our data therefore add into the current debate surrounding the merits of practising routine washing of NICU babies.

There are several limitations to our study. While both NICUs operated under similar clinical guidelines, inevitable differences in practices exist, and further work to study multiple centres with differing antiseptic regimes would be warranted. We did not include extensive genome sequencing of isolates and it is possible that different lineages of CoNS are established in each site, although our recent work comparing isolates from UK and Germany found this not to be the case [31]. The swab collection periods between the centres were not concurrent, though no significant potentially-confounding changes in units' practices occurred in the intervening period. The collection periods were also short and consequently data could not be analysed longitudinally. Also, a larger number of isolates from NNUH were included which may skew comparisons to some degree.

## Conclusion

In summary, this two-site observational study shows that frequent whole-body skin washing with OCT compared to not routinely washing does not appear to result in a lasting reduction in numbers of CoNS organisms found on the skin, therefore suggesting that the practice of daily OCT washing may be of limited clinical value in reducing blood culture-positive sepsis rates in the NICU. This hypothesis merits further study in a randomised controlled trial. Nevertheless, routine OCT washing does not appear to select for OCT-tolerant organisms in the short term. Isolates from the NNUH were much less susceptible to OCT and CHX antiseptics than were the BRI isolates, suggesting that the bathing of neonates in the BRI NICU does not select for resistance. The data suggest that different antiseptic regimes can have significantly different impacts on the microbiota in terms of both composition and antiseptic susceptibility.

Large-scale clinical trials to compare efficacy, safety and microbiological impacts of different antiseptic washing regimes and practices systematically are now required to design evidence-informed guidelines for this vulnerable patient group. Understanding how best to prevent neonatal bloodstream infection is vital in order to produce best clinical practice guidelines which will minimise invasive infection and the potential for selection of antiseptic resistance.

## Acknowledgements

We sincerely thank all NNUH and BRI research and clinical nurses who helped with skin swab collection. We are grateful to Julie Dawson, Research Services Manager at NNUH for reviewing our study protocol. We are most grateful to the anonymous reviewers for very constructive and helpful comments on an earlier version of our manuscript.

## Ethical statement

This study did not require any formal UK Research Ethics committee review or Health Research Authority approval because it did not meet contemporaneous criteria for NHS Research requiring such prior approvals. The Research Services Manager of the Norfolk and Norwich University Hospitals NHS Foundation Trust reviewed the study protocol prior to project commencement and confirmed that formal research ethics review was not required for this surveillance study.

## Availability of data and materials

Not applicable.

## Conflict of interest statement

The authors have no relevant financial or non-financial interest to disclose.

## Funding statement

This work was supported by an award from the 10.13039/501100000268Biotechnology and Biological Sciences Research Council (BB/T014644/1).

## CRediT author statement

**Heather Felgate:** Data curation; Formal analysis; Investigation; Validation; Visualization; Roles/Writing – original draft, review & editing.

**Charlotte Quinn:** Data curation; Investigation; Roles/Writing – review & editing.

**Ben Richardson:** Data curation; Investigation; Roles/Writing – review & editing.

**Carol Hudson:** Data curation; Investigation; Roles/Writing – review & editing.

**Dheeraj Sethi:** Data curation; Investigation; Roles/Writing – review & editing.

**Sam Oddie:** Investigation; Methodology; Project administration; Resources; Supervision; Validation; Writing – review & editing.

**Paul Clarke:** Conceptualization; Investigation; Methodology; Project administration; Resources; Supervision; Roles/Writing– review & editing, summary data analysis, final draft.

**Mark Webber:** Conceptualization; Data curation; Formal analysis; Funding acquisition; Investigation; Methodology; Project administration; Resources; Supervision; Validation; Visualization; Roles/Writing – original draft; Writing – review & editing.
